# The role of the transcription factor - Prospero homeobox 1 (Prox1) in the biology of differentiated thyroid cancer - the effect of the expression of Prox1 on migration and invasiveness of thyroid cancer cells

**DOI:** 10.1186/1756-6614-6-S2-A51

**Published:** 2013-04-05

**Authors:** Magdalena Rudzińska, Damian Gaweł, Anna Stachurska, Hanna Domek, Jolanta Czerwińska, Mirosław Kiedrowski, Tomasz Stępień, Dariusz Lange, Barbara Czarnocka

**Affiliations:** 1 Department of Biochemistry and Molecular Biology, The Centre of Medical Postgraduate Education, Warsaw, Poland; 2 Department of Immunohematology, The Centre of Medical Postgraduate Education, Warsaw, Poland; 3 Department of Pathomorphology, The Centre of Medical Postgraduate Education, Warsaw, Poland; 4 Maria Sklodowska-Curie Memorial Cancer Centre and Institute of Oncology, Warsaw, Poland; 5 Department of Endocrine and General Surgery, Medical University of Lodz, Lodz, Poland; 6 Maria Sklodowska-Curie Memorial Cancer Centre and Institute of Oncology, Gliwice, Poland

## 

The feature of malignancy is its ability to infiltrate and metastasis. The differentiated thyroid carcinoma (DTC), which constitutes of more than 80% of all thyroid cancers is spread by two major paths: papillary carcinoma (PTC) usually by the lymphatic vessels, and follicular cancer (FTC) mainly through the circulatory system. It was proposed that the cancers microenvironment induced by lymphangiogenesis could be an important factor affecting the expansion of primary tumour cells to lymph nodes.

The aim of our work was to investigate signaling pathways associated with migration and invasiveness of thyroid cancer cells. We were especially interested in the role of Prospero homeobox 1 (Prox1) protein, an important element of the lymphangiogenesis.

The studies were performed in a series of thyroid cancer FTC and PTC cell lines. The protein expression level and intracellular localization of the Prox1 gene was determined using molecular biology methods including: Q-RT-PCR, Western Blot and immunocytochemistry. The tumour cells' dynamics and invasiveness were monitored by microscopic observation assay and using invasion test, respectively. Additionally, the cells phenotype under Prox1 gene overproduction and silencing conditions was investigated.

We observed significant differences in the level of expression Prox1 between tested thyroid cancer cell lines. Additionally, protein subcellular localization was also variable and correlated with the tumour type. The protein concentration was not directly combined with mRNA level and even under small mRNA concentration in some tissue culture lines, the Prox1 protein was cumulated in the cells over the time. We noticed that the increased expression level of Prox1 gene resulted in a lower rate of cell migration. Interestingly, a similar effect was demonstrated under overexpression conditions of the Prox1 gene, but uniquely, the lower dynamic rate of cells was additionally accompanied by a significantly higher invasive potential of the tested cells.

The differences in the expression level and localization of Prox1 protein in various DTC lines may suggest an important role of it in the spreading of follicular and papillary carcinomas. We showed that Prox1 is a factor that has a significant effect on the migration and invasiveness of thyroid cancer cells, and thus affects the spread of the tumour. We propose that Prox1 may lead to changes in the signaling pathway controlling cytoskeleton dynamics in the tumour cells and the turnover of invasiveness. Further on-going studies will precisely determine the role of Prox1 lymphangiogenesis factor in DTC propagation.

